# Gemcitabine potentiates the anti-tumour effect of radiation on medullary thyroid cancer

**DOI:** 10.1371/journal.pone.0225260

**Published:** 2019-11-14

**Authors:** Viktor Sandblom, Johan Spetz, Emman Shubbar, Mikael Montelius, Ingun Ståhl, John Swanpalmer, Ola Nilsson, Eva Forssell-Aronsson

**Affiliations:** 1 Department of Radiation Physics, Institute of Clinical Sciences, Sahlgrenska Cancer Center, Sahlgrenska Academy, University of Gothenburg, Gothenburg, Sweden; 2 Department of Medical Physics and Biomedical Engineering, Sahlgrenska University Hospital, Gothenburg, Sweden; 3 Department of Pathology, Institute of Biomedicine, Sahlgrenska Cancer Center, Sahlgrenska Academy, University of Gothenburg, Gothenburg, Sweden; University of Wisconsin, UNITED STATES

## Abstract

Patients with medullary thyroid cancer (MTC) are often diagnosed with spread tumour disease and the development of better systemic treatment options for these patients is important. Treatment with the radiolabelled somatostatin analogue ^177^Lu-octreotate is already a promising option but can be optimised. For example, combination treatment with another substance could increase the effect on tumour tissue. Gemcitabine is a nucleoside analogue that has been shown to sensitise tumour cells to radiation. The aim of this study was to investigate potentially additive or synergistic effects of combining radiation with gemcitabine for treatment of MTC. Nude mice transplanted with patient-derived MTC tumours (GOT2) were divided into groups and treated with radiation and/or gemcitabine. Radiation treatment was given as ^177^Lu-octreotate or external beam radiotherapy (EBRT). The volume of treated and untreated tumours was followed. The absorbed dose and amount of gemcitabine were chosen to give moderate tumour volume reduction when given as monotherapy to enable detection of increased effects from combination treatment. After follow-up, the mice were killed and tumours were immunohistochemically (IHC) analysed. Overall, the animals that received a combination of EBRT and gemcitabine showed the largest reduction in tumour volume. Monotherapy with EBRT or gemcitabine also resulted in a clear detrimental effect on tumour volume, while the animals that received ^177^Lu-octreotate monotherapy showed similar response as the untreated animals. The GOT2 tumour was confirmed in the IHC analyses by markers for MTC. The IHC analyses also revealed that the proliferative activity of tumour cells was similar in all tumours, but indicated that fibrotic tissue was more common after EBRT and/or gemcitabine treatment. The results indicate that an additive, or even synergistic, effect may be achieved by combining radiation with gemcitabine for treatment of MTC. Future studies should be performed to evaluate the full potential of combining ^177^Lu-octreotate with gemcitabine in patients.

## Introduction

Medullary thyroid cancer (MTC) accounts for about 1–2% of all thyroid cancers [1]. It originates from the calcitonin-producing parafollicular C-cells of the thyroid and occurs either sporadically or as a hereditary form in the multiple endocrine neoplasia type 2 syndrome, often caused by mutations in the *RET* proto-oncogene [2–4]. Many patients with MTC present with metastatic disease at the time of diagnosis and curative surgery can only be performed in patients with limited or no tumour spread to local lymph nodes [5, 6]. Based on 1252 cases between 1973 and 2002 registered in the Surveillance, Epidemiology, and End Results (SEER) database, the 10-year survival for patients with MTC confined to the thyroid gland is about 95% compared with 40% for patients diagnosed with distant metastases [7]. Therefore, there is a need for better systemic therapy strategies for metastatic disease.

For distant metastases from the more common papillary and follicular thyroid cancer, systemic therapy with radioiodine (^131^I) is a well-established treatment technique with high response rates [8]. However, since MTC originates from the C-cells of the thyroid, it lacks the transmembrane protein NIS (sodium/iodine symporter) that is responsible for transporting iodide into the cell, and MTC can therefore not be treated with radioiodine. Instead, many MTCs express somatostatin (SST) receptors (SSTRs), which is a characteristic feature also for other types of neuroendocrine tumours (NETs) [9]. Therefore, high receptor-specific binding of radiolabelled SST analogues, *e*.*g*. ^111^In-octreotide or ^177^Lu-octreotate, can be achieved in MTC [10, 11]. Treatment with radiolabelled SST analogues is one type of peptide receptor radionuclide therapy (PRRT). In a clinical phase II trial, PRRT with ^90^Y-octreotide for patients with metastatic MTC was associated with long-term survival benefit [12]. However, few patients are cured with the current standardised treatment protocol. There is a clear need for optimisation and one option could be to combine PRRT with another drug [13].

Gemcitabine is a nucleoside analogue that has shown anti-tumour activity in many different cancer types, including MTC [14, 15]. After entering a cell, gemcitabine is activated by phosphorylation and the active metabolites gemcitabine diphosphate and triphosphate are generated. These metabolites are responsible for the cytotoxic effect by 1) incorporation in the DNA, which inhibits DNA polymerases, leading to G1/S cell cycle arrest or cell death, and by 2) interfering with the enzyme ribonucleotide reductase responsible for DNA synthesis and repair [16].

Chemotherapeutic drugs can enhance the cell-killing effect of radiation, a process called radiosensitisation [17]. In preclinical studies, gemcitabine has a well-documented radiosensitising effect on many different cancer types [18–22]. Furthermore, several clinical studies have evaluated the use of gemcitabine in combination with external beam radiotherapy (EBRT), most frequently for pancreatic cancer [23–27]. For treatment of metastatic MTC, it is of value to investigate the efficiency of the combination of gemcitabine and radiation, both as EBRT and as systemic radionuclide therapy using radiolabelled SST analogues, *e*.*g*. ^177^Lu-octreotate. To the authors’ knowledge, no such investigations have yet been made.

The aim of this study was to investigate the potentially additive or synergistic effects of combining ionising radiation (^177^Lu-octreotate and EBRT) with gemcitabine for treatment of MTC. The study was performed in MTC-bearing mice.

## Material and methods

### Radiopharmaceutical

^177^Lu-octreotate was purchased from IDB Holland (IDB Holland BV, Baarle-Nassau, the Netherlands). Radiolabelling was performed according to the manufacturer’s instructions. Instant thin layer chromatography (ITLC) was used to measure the amount of peptide bound ^177^Lu, which was determined to be over 99%. All syringes containing ^177^Lu-octreotate were measured in a well-type ionisation chamber (CRC-15R, Capintec, Ramsey, New Jersey, USA) before and after injection to determine the amount of injected radiopharmaceutical into each animal.

### Animal model

GOT2 are MTC cells that have been successfully transplanted to nude mice [28]. Originally, MTC cells were collected from a patient with sporadic, *RET*-driven MTC in Gothenburg. By serially transplanting GOT2 tumours to new generations of mice, we have used this patient-derived xenograft model for studies of MTC for over 15 years. The collection of tumour tissue from the patient was performed during surgery in 2001. At that time, formal ethical approval by an ethics committee was optional according to Swedish law, but was in this case not regarded necessary. The patient provided oral informed consent for the collection according to standard procedure at the time. The consent was verified by two surgeons, one pathologist and one medical physicist. The principles expressed in the Declaration of Helsinki was followed.

In this study, small GOT2 tumour tissue samples (*ca*. 1x1x1 mm^3^) were transplanted subcutaneously in the neck of 4–5 weeks old female BALB/c nude mice (Charles River Laboratories, Sulzfeld, Germany) under anaesthesia by intraperitoneal (*i*.*p*.) injection of Ketaminol® vet. (Intervet AB, Stockholm, Sweden) and Domitor® vet. (Orion Pharma AB Animal Health, Sollentuna, Sweden). An *i*.*p*. injection of Antisedan® vet. (Orion Pharma AB Animal Health) was used as antidote to anaesthesia. After about 2 months, tumours appeared close to the transplanted location. Digital callipers were used to measure the tumour length, width, and height. Then, the tumour volume was calculated by assuming an ellipsoidal shape. The experiments were initiated when the tumours reached a volume of about 200–2000 mm^3^ (mean = 570 mm^3^, SD = 406 mm^3^). All mice were given water and autoclaved food *ad libitum*. The experiments were approved by the Ethical Committee on Animal Experiments in Gothenburg, Sweden (permit no. 107–15).

### Combination therapy experiments

GOT2-bearing mice (n = 48) were divided into groups of 5–12 mice per group. The tumour size distribution within each group was kept as similar as possible. Three groups were used for combination studies of ^177^Lu-octreotate and gemcitabine, another three groups were used for combination studies of EBRT and gemcitabine, and one group was used as untreated control animals (Table 1). The ^177^Lu activity, absorbed dose, and amount of gemcitabine were chosen to give low to moderate tumour volume reduction as monotherapy to enable detection of any additive or synergistic effects in the combination therapy groups.

**Table 1:** GOT2-carrying nude mice were treated according to the presented schedules with ^177^Lu-octreotate (Lu), gemcitabine (Gem) and/or external beam radiotherapy (EBRT), or left untreated as control animals.

		Administered amount: Lu (MBq), Gem (mg/kg) or EBRT (Gy)						
Group	T (d)	Day 0	Day 3	Day 7	Day 10	Day 13	n	F (d)
Lu	96	10	0	0	0	0	5	16
Gem high	96	125	125	125	60	60	5	16
Lu + Gem high	96	10+125	0+125	0+125	0+60	0+60	5	16
EBRT	62	5	0	0	0	0	5	44
Gem low	137	60	60	60	60	60	10	44
EBRT + Gem low	62	5+60	0+60	0+60	0+60	0+60	6	58
Untreated control	145	-	-	-	-	-	12	30

T, median time between transplantation and treatment start; n, number of animals in each group; F, follow-up time.

^177^Lu-octreotate (10 MBq, 0.1 ml) was administered by intravenous injection in the tail vein as a single treatment on day 0. Gemcitabine (Active Biochem LTD, Hong Kong, China) was obtained as a powder, which was dissolved in saline solution, and 60 or 125 mg/kg was administered by *i*.*p*. injection twice a week for a total of 2.5 weeks starting on day 0. Prior to each injection of gemcitabine, the mice were weighed and the injected volume (0.15–0.20 ml) was adjusted to administer the correct dose to each mouse. No symptoms of toxic side effects were seen for the animals receiving ^177^Lu-octreotate. However, the initial twice weekly gemcitabine dose of 125 mg/kg for the groups used for combination treatment studies of ^177^Lu-octreotate and gemcitabine resulted in weight loss for many animals. Therefore, the gemcitabine dose was lowered to 60 mg/kg from day 10 for these animals. Unfortunately, this change did not improve the condition of the animals and this treatment group was only followed for 16 days (Table 1). For the same reason, the animals used for combination treatment studies of EBRT and gemcitabine received 60 mg/kg from day 0.

For EBRT, the animals were anaesthetised (by *i*.*p*. injection of Ketaminol® vet. and Domitor® vet.), placed on their side on a tissue-equivalent polystyrene bed and individually irradiated using a Varian linear accelerator with 6 MV photon beam (nominal energy) at a gantry angle of 0 degrees (Varian Medical Systems, Palo Alto, California, USA). To obtain a relatively uniform dose distribution in the tumour and to minimise air gaps, tissue-equivalent material was fitted around the mouse and the tumour. The tumour was covered with 15 mm tissue-equivalent material and the centre of the tumour was positioned at isocenter at a depth of about 20 mm (depending on the size of the tumour). A 30x30 mm^2^ irradiation field was then used to deliver 5 Gy to the tumour. Using this setup, adjacent normal tissues were irradiated to some extent (although with lower exposure than in other positioning). However, the animals showed no apparent signs of side effects from the EBRT during the follow-up time of the animals.

### Internal dosimetry

For ^177^Lu-octreotate exposure, estimations of mean absorbed dose, *D*, were made according to the Medical Internal Radiation Dose (MIRD) formalism [29]:

$$D=\frac{Ã\sum_{i}E_{i}Y_{i}\phi_{i}}{M}$$ (1)

where *Ã* is the time-integrated activity, the product *E*_*i*_*Y*_*i*_ is the energy emitted per decay for the *i*^th^ nuclear transition, *ϕ*_*i*_ is the absorbed fraction and *M* is the mass of the tissue of interest. Only the contribution from beta particles was considered. Therefore, *Σ*_*i*_*E*_*i*_*Y*_*i*_ was set to 147.9 keV and the absorbed fraction was set to 1 [30, 31]. The time-integrated activity from time of injection to infinity time was estimated based on previously published biodistribution data of ^177^Lu-octreotate in BALB/c nude mice carrying GOT2 tumours, by fitting a mono-exponential curve to the time-activity concentration data [32].

### Post-treatment follow-up

After start of treatment, the animals were monitored and tumour volume was measured twice a week. For each mouse, the relative tumour volume was defined as the tumour volume at a given point-in-time divided by the tumour volume at day 0 (start of treatment). Each group was followed until most of the tumours in a group had regrown (relative tumour volume at least larger than 1, but usually *ca*. 3–4). In addition, a mouse that met one of the following criteria was killed: 1) the tumour reached a volume corresponding to more than 10% of the total body weight, 2) the body weight was reduced by more than 10%, or 3) the mice showed any signs of lower health status. The mice were killed by cardiac puncture under anaesthesia (Pentobarbitalnatrium vet., Apotek Produktion & Laboratorier AB, Huddinge, Sweden) and tumours were fixed in formalin for histological studies. The follow-up time for each group can be seen in Table 1.

### Immunohistochemistry

The formalin-fixed tumours were embedded in paraffin and processed for histological examination by standard procedures. The tumours were sliced into 4-μm sections and stained with haematoxylin and eosin (H&E) and Masson’s trichrome (MT) for examination of morphology and fibrosis, respectively.

For immunohistochemical (IHC) analysis, tumour sections were placed on glass slides and treated with EnVision^™^ FLEX Target Retrieval Solution (high pH) using a PT-Link (Dako, Glostrup, Denmark). The IHC staining was performed using an Autostainer Link using EnVision^™^ FLEX according to the manufacturer’s instructions (Dako). In each run, positive and negative controls were included. To analyse cell proliferation, a primary antibody for Ki67 (AB9260, Merck Millipore, Burlington, Massachusetts, USA) was used. To verify MTC origin of the tumours, tumour tissue sections from the untreated control group were stained using antibodies for MTC markers chromogranin A (ab68271, Abcam, Cambridge, England), synaptophysin (ab16659, Abcam), and calcitonin (A0576, Dako). Imaging for figure presentation of MTC markers was performed using a microscope (20x magnification, Eclipse E1000, Nikon Instruments, Amsterdam, Netherlands) equipped with a camera (ProgRes C7, Jenoptik, Jena, Germany).

Tumour sections stained for Ki67 and MT were digitalised using a digital slide scanner at 40x magnification (Leica SCN400 Slide Scanner, Leica Microsystems, Germany). Then, quantitative analysis of histological features were performed on these digitalised sections (resolution 0.25x0.25 μm^2^) using an in-house developed MATLAB tool (R2017a, MathWorks, Natick, Massachusetts, USA). In brief, tumour tissue was delineated by drawing a region of interest (ROI) around the tumour, excluding the tumour capsule. Thereafter, colour thresholds were applied to segment and remove regions of cracks (background) or folds caused by histological processing, and to segment positively stained tumour tissue. Necrotic tumour regions were easily discernible in the MT images (and verified on H&E sections), which facilitated automatic segmentation of tumour tissue into necrotic or non-necrotic tumour (denoted as viable). By aligning the digital Ki67 and MT sections by semi-automatic image registration (MATLAB control point selection tool), the segmentation mask for viable/necrotic tumour tissue defined from MT staining was applied also to the Ki67 sections. The colour thresholds and corresponding segmentation of tumour tissues were approved by a board-certified pathologist (O.N.). Ki67 was quantified only in viable tumour, whereas MT (blue regions representing collagen) was quantified for the entire tumour section (excluding background and artefacts).

### Data calculations and statistical analyses

The relative tumour volume was calculated individually for each mouse. Then, mean relative tumour volumes for each group was calculated and used for the statistical analyses. Group differences were analysed for all measurements after study start when all groups were still followed (*i*.*e*., day 3–16 or day 3–30, Table 1). For each of these measurements, overall differences between all groups were analysed using one-way ANOVA in GraphPad Prism 7.04 (GraphPad Software, La Jolla, San Diego, California, USA). Furthermore, group-to-group differences were analysed using Student’s t-tests. All reported p-values were adjusted to account for multiple comparisons using Bonferroni-Holm correction [33]. An adjusted p-value of less than 0.05 was considered statistically significant for all tests.

The interaction effect between radiation and gemcitabine was analysed using the Bliss independence model [34]. Based on the mean relative tumour volumes, the predicted additive fractional response for a given combination, F_add_, was calculated using

$$F_{add}=F_{rad}+F_{gem}-F_{rad}F_{gem}$$ (2)

where F_rad_ and F_gem_ is the fractional response from monotherapy with radiation (^177^Lu-octreotate or EBRT) and with gemcitabine, respectively [35]. The fractional response was calculated using

$$F=1-\frac{{RTV}_{mono}}{{RTV}_{control}}$$ (3)

where RTV_mono_ and RTV_control_ is the mean relative volume in a monotherapy group and in the untreated control group, respectively. Lastly, F_add_ was compared with the measured effect in the combination therapy group. The measured effect was assumed to be synergistic, additive or antagonistic if it was larger than, equal to or smaller than F_add_, respectively.

## Results

### Anti-tumour effects of ^177^Lu-octreotate and/or gemcitabine

**Fig 1:**
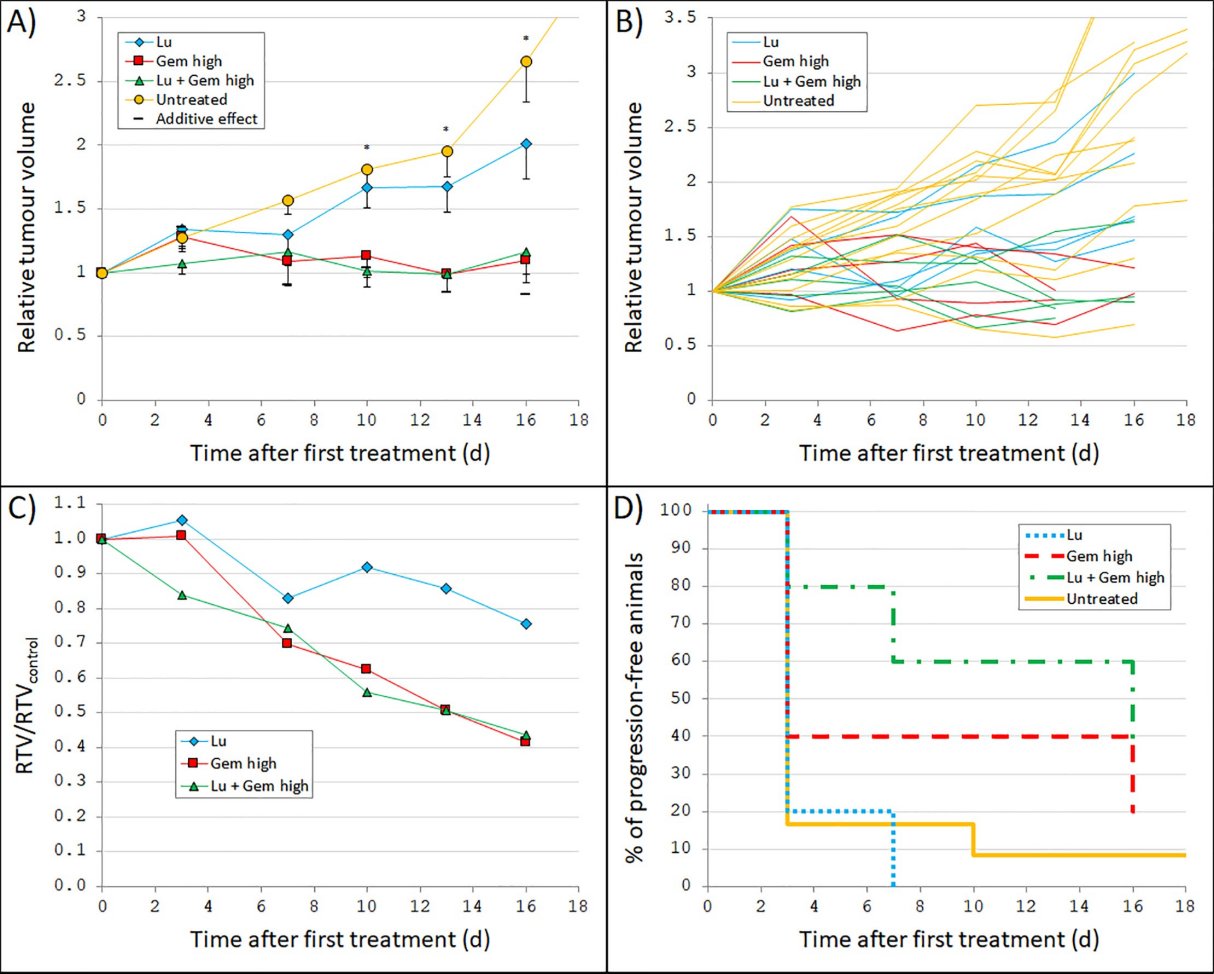
Tumour growth after ^177^Lu-octreotate and/or gemcitabine treatment. GOT2-carrying nude mice were treated with 10 MBq ^177^Lu-octreotate (Lu) and/or 125 mg/kg (initially, but lowered to 60 mg/kg) gemcitabine twice weekly (Gem high). The ^177^Lu activity and amount of gemcitabine were chosen to give low to moderate effect as single treatment to enable detection of any additive or synergistic effects. **(A)** Mean relative tumour volume (RTV) *vs*. time after first treatment. In addition to the measured values for each group, also the calculated relative tumour volume given a predicted additive response (Eq (2)) is shown. Error bars show SEM. A star indicate that the ANOVA analyses (performed at day 3–16) resulted in a statistically significant difference between the group means. **(B)** RTV *vs*. time after first treatment for each individual mouse. **(C)** Mean RTV for each treatment group divided by mean RTV for the untreated control group (RTV/RTV_control_) *vs*. time after first treatment. **(D)** Percentage of animals in each group without tumour progression *vs*. time after first treatment. Not all animals reached tumour progression before the end of follow-up, and therefore, some lines end at values higher than 0%. The line for the untreated control animals continues past 16 days because the follow-up time was longer for these animals. Note differences in scale of the x-axis compared with Fig 2.

The administered activity of 10 MBq ^177^Lu-octreotate in this study resulted in a mean absorbed dose to tumour of 0.13 Gy. Tumour volume *vs*. time curves for nude mice carrying the patient-derived MTC model GOT2 treated with 10 MBq ^177^Lu-octreotate and/or 125 mg/kg gemcitabine (125 mg/kg initially but lowered to 60 mg/kg) are shown in Fig 1A–1C. ANOVA analyses of the relative tumour volumes revealed statistically significant overall differences between all four groups at day 10–16 (p = 0.428, 0.071, 0.009, 0.006, and 0.049 at day 3, 7, 10, 13, and 16, respectively). Absolute tumour volumes (mm^3^) are provided in S1 Fig.

Given as monotherapy, gemcitabine resulted in a detrimental effect on tumour growth. At day 13, the relative tumour volume for the animals treated with gemcitabine was 51% of the relative volume for the untreated control animals (p = 0.045, Fig 1A). Furthermore, the animals that received only ^177^Lu-octreotate showed similar response as the untreated control animals (p = 0.249 at day 16). Consequently, the animals treated with a combination of both ^177^Lu-octretoate and gemcitabine showed no statistically significant difference in tumour volume compared with the animals treated with gemcitabine only (p = 0.430 at day 16).

In Fig 1D, Kaplan-Meier curves of progression-free survival (PFS) are shown. Tumour progression was defined as occurring when a tumour was larger than at start of treatment (after initial treatment response) or when an animal was killed. The longest time to progression (TTP) was seen for the combination therapy group, while the ^177^Lu-octreotate group showed similar PFS as the untreated control group, and gemcitabine monotherapy resulted in a prolonged TTP. For example, two weeks after start of treatment, about 90% of the control animals and all ^177^Lu-octreotate-treated animals had reached tumour progression, with corresponding values of 60% and 40% for the gemcitabine and combination therapy groups, respectively.

### Anti-tumour effects of EBRT and/or gemcitabine

**Fig 2:**
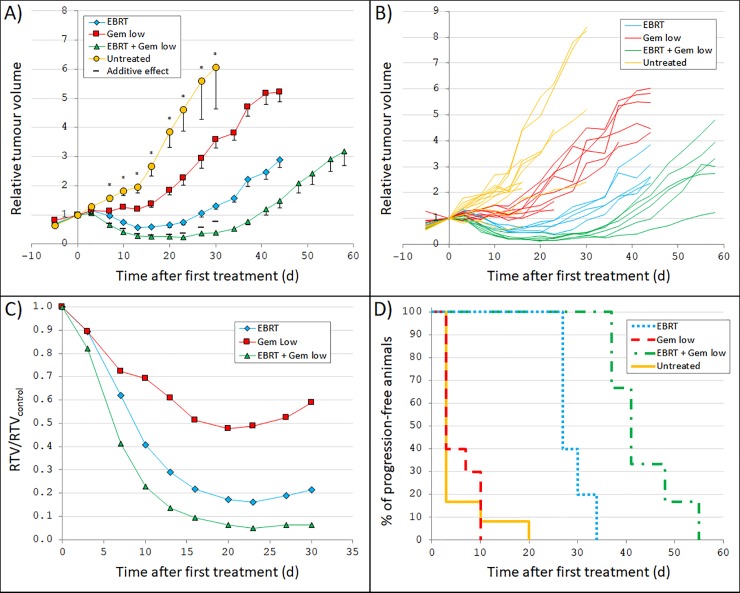
Tumour growth after external beam radiotherapy and/or gemcitabine treatment. GOT2-carrying nude mice were treated with 5 Gy external beam radiotherapy (EBRT) and/or 60 mg/kg gemcitabine twice weekly (Gem low). The absorbed dose and amount of gemcitabine were chosen to give low to moderate effect as single treatment to enable detection of any additive or synergistic effects. **(A)** Mean relative tumour volume (RTV) *vs*. time after first treatment. In addition to the measured values for each group, also the calculated relative tumour volume given a predicted additive response (Eq (2)) is shown. Error bars show SEM. A star indicate that the ANOVA analyses (performed at day 3–30) resulted in a statistically significant difference between the group means. **(B)** RTV *vs*. time after first treatment for each individual mouse. **(C)** Mean RTV for each group divided by mean RTV in the untreated control group (RTV/RTV_control_) *vs*. time after first treatment. **(D)** Percentage of animals in each group without tumour progression *vs*. time after first treatment. Note differences in scale of the x-axis compared with Fig 1.

Tumour volume *vs*. time curves for the animals treated with 5 Gy EBRT and/or 60 mg/kg gemcitabine can be seen in Fig 2A–2C. ANOVA analyses revealed statistically significant overall differences between all four groups at all follow-up measurements, except for at 3 days after treatment (p = 0.156 at day 3, and p<0.001 at day 7–30). Absolute tumour volumes (mm^3^) are provided in S1 Fig.

Given as monotherapy, both gemcitabine and EBRT resulted in a clear detrimental effect on tumour growth. Compared with the tumour volume in the untreated control group, the largest effect of the gemcitabine treatment was seen after 20 days when the relative volume was 48% of the relative volume in the untreated group (p<0.001). For the animals treated with EBRT, a clear initial tumour volume reduction was seen. Seven days after treatment, the mean relative tumour volume was 0.97 and reached a minimum after 13 days when the relative volume was 0.57, corresponding to 29% of the relative volume in the untreated group (p<0.001). Thereafter, the tumours started to regrow with a growth rate similar to what was seen in the gemcitabine group and in the untreated group (Fig 2A).

Overall, the animals that received a combination of EBRT and gemcitabine showed the largest reduction in tumour volume over time of all groups (Fig 1–Fig 2). One week after start of treatment, the relative volume was 0.65 and reached a minimum of 0.23 at 23 days after treatment start, corresponding to only 5% of the relative volume in the untreated control group (p<0.001). Furthermore, the analyses of interaction effects showed that the treatment effect for these animals was larger than the calculated predicted additive response for several measurements after start of treatment (Fig 2A).

The PFS curves showed a substantially prolonged TTP for the animals treated with EBRT monotherapy, with a median TTP of 27 days (Fig 2D). However, the TTP for the gemcitabine-treated animals was similar to that of the untreated control group, with a median TTP of 3 days for both groups. Overall, the animals that received a combination of EBRT and gemcitabine showed the longest TTP of all groups, with a median TTP of 41 days (Fig 2D). Altogether, the results show that gemcitabine clearly enhances the anti-tumour effect of radiation therapy on GOT2.

### Immunohistochemical analyses

**Fig 3:**
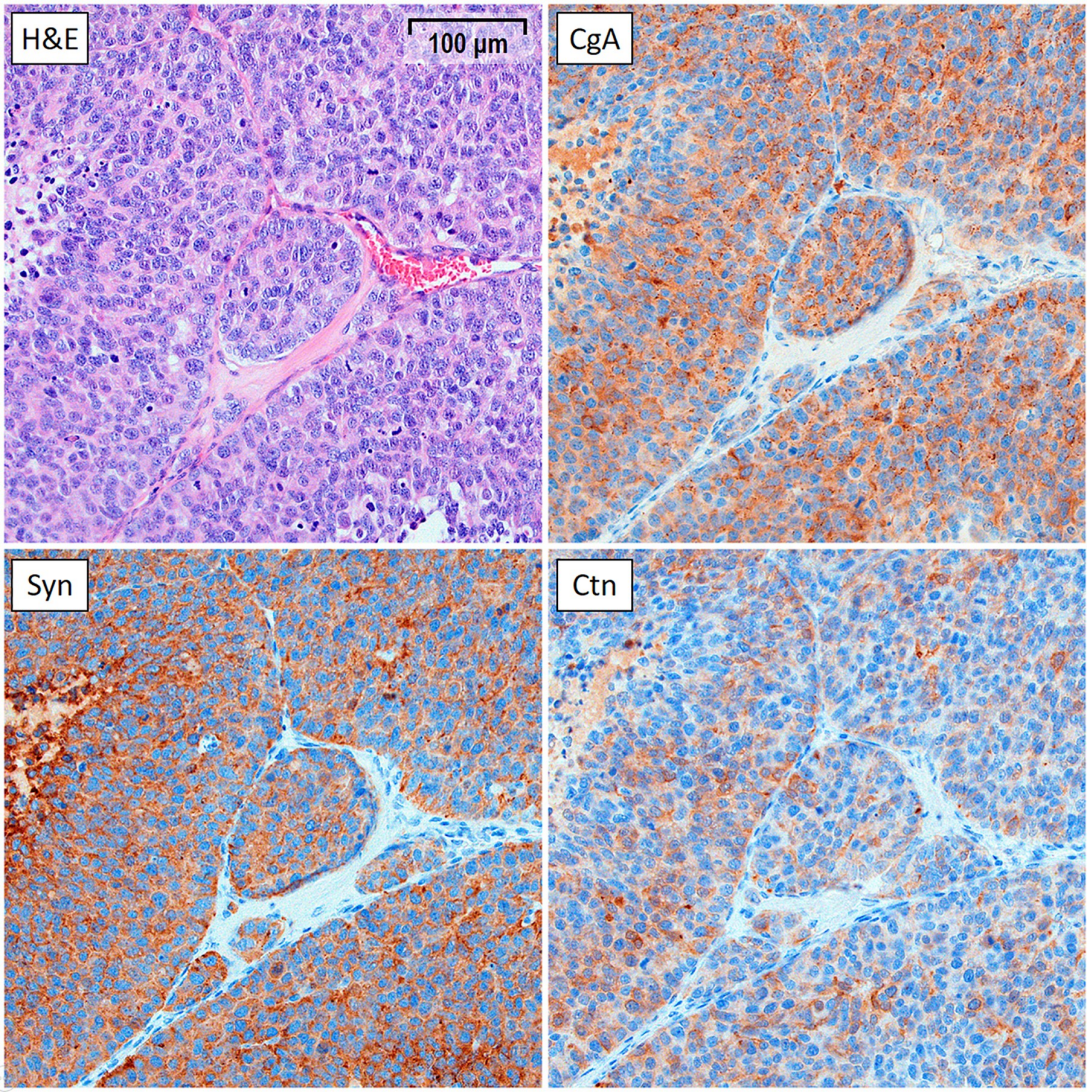
IHC staining of a representative GOT2 tumour (20x magnification). The tumour was harvested from an animal in the untreated control group 30 days after start of treatment. The growth curve for this tumour was similar to the mean growth curve of the control group. The tumour was stained with haematoxylin and eosin (H&E), as well as for MTC markers chromogranin A (CgA), synaptophysin (Syn), and calcitonin (Ctn).

The stained tumours in the untreated control group showed a high and specific expression of MTC markers chromogranin A, synaptophysin, and calcitonin, and were morphologically consistent with MTC (Fig 3).

**Fig 4:**
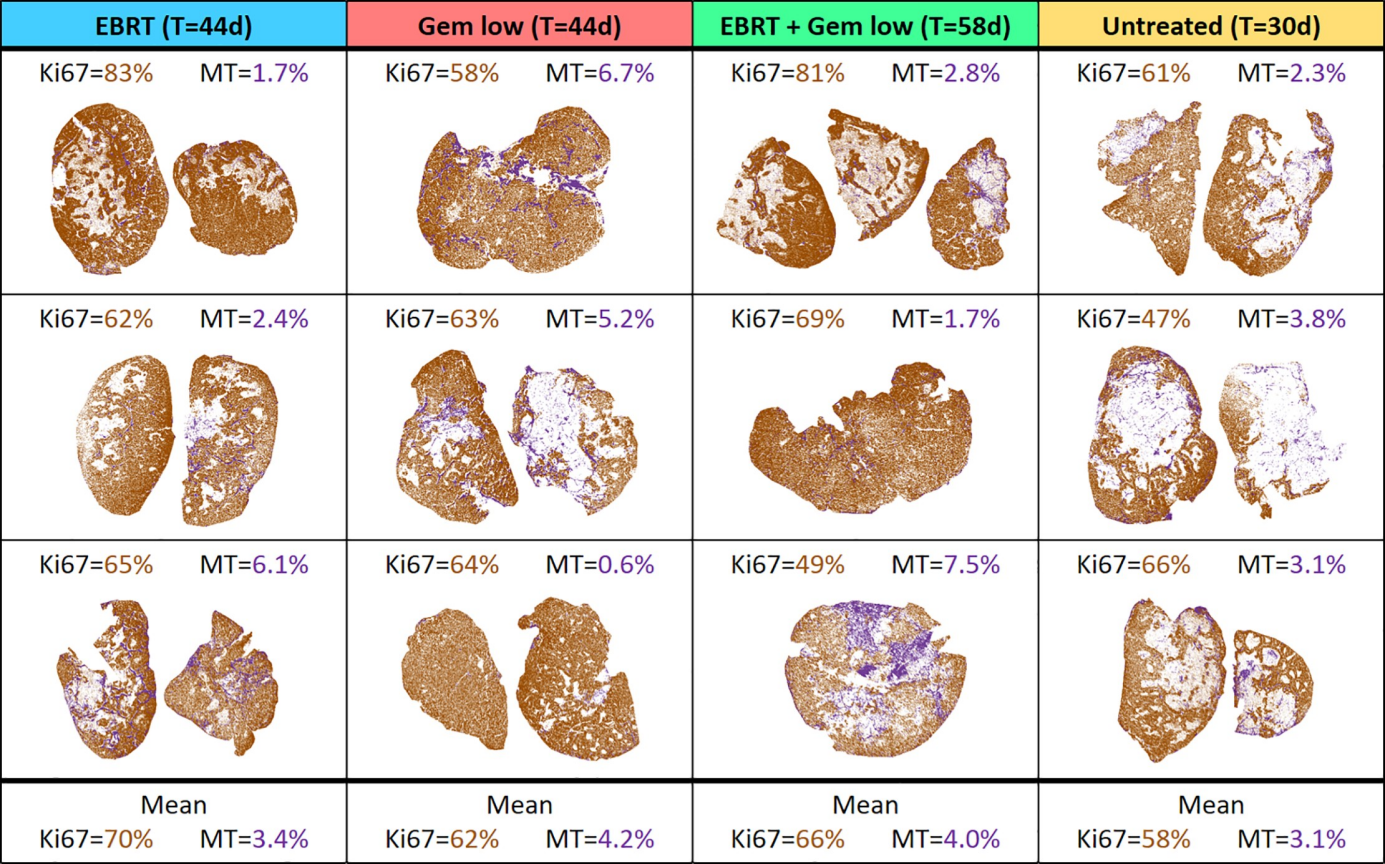
Overview of the results of the quantitative IHC analyses. Analyses were performed for the animals treated with 5 Gy external beam radiotherapy (EBRT) and/or 60 mg/kg gemcitabine (Gem low) as well as for the untreated control animals. Quantification was made for digitalised histological sections stained with Ki67 (shown in brown) and Masson’s trichrome (MT, shown in purple). For each tumour, the percentage of the area positively stained for Ki67 (in viable tumour only) and MT (in the whole tumour area) was determined. Included is also the follow-up time, T, for each group.

The percentage of Ki67-positive tissue (in viable tumour areas) was similar in all groups with an overall median of 64% (Fig 4). The percentage of MT-positive tissue (in whole tumour) was also similar in all groups, with an overall median of 3.0%. However, several tumour sections from mice that received radiation and/or gemcitabine treatment showed higher percentage of MT-positive staining (higher than 6%) compared with the untreated control where all values were similar to the overall median.

## Discussion

In this study, we have investigated the therapeutic effect of combining ionising radiation with gemcitabine for treatment of medullary thyroid cancer (MTC). Using a patient-derived MTC animal model (GOT2), we have provided results that indicate that an additive, or possibly even synergistic, combinatorial effect could be achieved. Furthermore, given as monotherapy, both gemcitabine and irradiation appeared to have a clear detrimental effect on tumour volume. It should be noted that suboptimal monotherapy doses and treatment protocols were used in order to enable detection of an increased effect from the combination treatment. Higher doses or a more optimal treatment protocol, *e*.*g*. repeated or differently scheduled treatment, would probably have resulted in a much higher effect on tumour volume in each of the monotherapy groups.

For the combination of irradiation and gemcitabine to be of value for patients with metastatic disease, systemic radionuclide therapy is needed (*e*.*g*. ^177^Lu-octreotate) instead of EBRT. Even though the ^177^Lu-octreotate monotherapy group showed similar response as the untreated control group in this study, previous data indicate that ^177^Lu-octreotate treatment is an option for patients with metastatic MTC with high SSTR expression [10, 11]. The biodistribution of ^177^Lu-octreotate in GOT2 nude mice has previously been studied [32]. The tumour uptake was comparable to what has been seen for radiolabelled SST analogues in patients with MTC, with a ratio of activity concentration in tumour and blood (T/B) of about 50 [10, 11]. However, there is a large individual variation between patients, and some patients have also shown much higher T/B values of up to 350, and these patients should be the most suitable for ^177^Lu-octreotate treatment. Furthermore, it is possible that the administered amount of 10 MBq ^177^Lu-octreotate used in this study was too low (absorbed dose to the GOT2 tumours was only 0.13 Gy). In a similar study, where ^177^Lu-octreotate was evaluated for treatment of small-intestine NETs in a xenograft animal model (GOT1), a clear effect on tumour volume was seen after administration of 15 MBq ^177^Lu-octreotate (resulting in 2.7 Gy to the tumour) [36, 37]. Since EBRT gave such high radiobiological effect, we believe that the poor response for the animals that received ^177^Lu-octreotate monotherapy could have been significantly improved if a higher absorbed dose would have been achieved. Because of the relatively low SSTR-expression in the GOT2 tumours, it would have been difficult to increase the absorbed dose by simply increasing the administered activity, due to tumour SSTR saturation. Unfortunately, there is, to our knowledge, no other MTC animal model available with higher SSTR-expression to better reflect the high uptake seen in some MTC patients; GOT2 is, as far as we know, the model with the highest uptake [32]. Therefore, we used EBRT in this study to be able to deliver a higher absorbed dose to the tumours and show that MTC is sensitive to irradiation.

Combination therapy offers many potential benefits for patients and can still be warranted even if there is no synergistic, but instead an additive or even antagonistic, effect between the drugs combined [38]. Firstly, if two drugs with non-overlapping toxic effects are used, the total administered amount can be increased while still keeping side effects below acceptable limits for the patient. Thus, the non-specific toxicity produced by a high dose of a single drug can be reduced. Another reason for using combination therapy is that a synergistic interaction effect on tumour tissue between the two drugs can occur if chosen wisely, increasing the tumour-killing potential. Furthermore, if a tumour develops resistance against the first drug, which is a common phenomenon in cancer therapy, the second drug can be an important next treatment step for the patient. It is also possible that, if administered at the same time, one of the drugs inhibits the development of resistance to the other drug. Lastly, both within-patient tumour heterogeneity and patient-to-patient variability are two additional arguments to use combination therapy. Within-patient tumour heterogeneity includes both differences within a given tumour and differences between two tumours, *e*.*g*. a primary tumour and its metastases, and in both situations, using more than one drug may be crucial to be able to target different populations of tumour cells and kill all cancer cells in the tumours if some cancer cells are not affected by only one drug. Furthermore, given a patient-to-patient variability, the use of drug combinations give each individual patient a better chance that at least one drug will be effective [39].

The results in this study show that an additive, or possibly even synergistic, effect could be achieved when combining irradiation and gemcitabine for treatment of MTC. A synergistic interaction effect could be explained by the mechanism of action of gemcitabine. By acting as a nucleoside analogue of deoxycytidine, gemcitabine can inhibit DNA synthesis and cause cell death [16]. Through this incorporation into the DNA, gemcitabine can also inhibit DNA repair of genomic damage caused by the radiation, leading to a theoretical interaction effect between irradiation and gemcitabine. Additionally, gemcitabine interferes with the enzyme ribonucleotide reductase, which is involved in DNA synthesis and repair. Lastly, there are also data suggesting that SST receptor (SSTR) expression can be upregulated by gemcitabine, further increasing the potential of radiolabelled SST analogue therapy for patients with metastatic MTC [40, 41]. This could be very useful if gemcitabine is administered before each injection of ^177^Lu-octreotate and would be interesting for future investigations.

The IHC analyses showed high expression of all MTC markers and a morphology consistent with that of MTC, verifying the MTC origin of the GOT2 tumours. The percentage of viable tumour area positively stained for Ki67 (indicating proliferative activity) was similar in all groups. This can be explained by the fact that the tumours were harvested for histological analysis at the end of follow-up (day 30–58), when all tumours had started to regrow. The amount of MT staining was also similar on a group level. However, some tumours in the treatment groups had a higher percentage of area positively stained for MT compared with untreated tumours. High amounts of positive MT staining indicates high levels of fibrosis, which in turn could be a sign of scarring due to treatment-related effects.

The experience of using gemcitabine as a treatment for patients with MTC is limited. In a study where gemcitabine (1000 mg/m^2^) was used to treat two patients with MTC, no apparent effect was seen [42]. However, for other cancer types, gemcitabine has been widely used for many years. In 1996, the U.S. Food and Drug Administration (FDA) approved gemcitabine for treatment of pancreas cancer and non-small cell lung cancer. Since then, it has also been approved for treatment of bladder, ovarian and breast cancer in combination with other drugs. The recommended dose of gemcitabine is 1000 mg/m^2^ on days 1 and 8 of each 21 day cycle [43]. At this dose level, gemcitabine is associated with side effects and among the most common (≥20% of patients) are nausea, vomiting, anemia, hepatic transaminitis, neutropenia, increased alkaline phosphatase, proteinuria, fever, hematuria, rash, thrombocytopenia, dyspnea, and peripheral edema. At lower doses, gemcitabine is less toxic and when used as a radiosensitiser rather than single-treatment agent, it is possible that much lower doses than 1000 mg/m^2^ can be used, minimizing the side effects while still increasing the therapeutic effect on tumour tissue. In a study on locally advanced head and neck cancer, gemcitabine appeared to be a strong radiosensitiser at doses much lower than those required for cytotoxic effects [24]. The amount of gemcitabine used in the present study was based on previous studies of gemcitabine in animal models, but also chosen to give low to moderate effect as monotherapy to enable the detection of any increased effect when the treatment was combined with irradiation. The dose of 60 mg/kg used for the mice in this study would correspond to about 180 mg/m^2^ for humans [44], which is considerably lower than the recommended 1000 mg/m^2^. Therefore, we believe that patients with MTC could benefit from the suggested combination therapy, while still being able to limit the dose of gemcitabine to keep side effects at an acceptable level.

The clinical experience of PRRT (*e*.*g*. ^177^Lu-octreotate or ^90^Y-octreotide) for MTC is relatively limited but larger than that of gemcitabine for MTC [12, 45–49]. Treatment outcomes differ between studies, but generally, response rates were around 40–70% with an estimated prolonged median survival of about 1–2 years together with very few side effects. It should be mentioned that these studies evaluating PRRT for MTC included relatively few patients. For other more common types of NET, PRRT has been widely used for over a decade and in studies including a much larger number of patients, PRRT has proven to be an efficient and safe treatment associated with few side effects [50–52]. However, few patients are cured with the current standard treatment protocol. An increased amount of administered activity should result in better treatment results but probably also a higher incidence of side effects. In recent years, ^177^Lu-octreotate has become more commonly used than ^90^Y-octreotide and in 2017 and 2018, respectively, the European Medicines Agency (EMA) and the FDA, approved the use of ^177^Lu-octreotate for the treatment of gastroenteropancreatic NETs.

## Conclusions

The results from this study showed additive, or even synergistic, therapeutic effects in MTC-bearing nude mice after radiation and gemcitabine treatment. The results indicate that it can be possible to achieve similar combinatorial effects also in patients with MTC. Future studies should be performed to evaluate the full potential of using ^177^Lu-octreotate for treatment of highly SSTR-expressing MTCs, both as monotherapy and in combination with gemcitabine.

## Supporting information

S1 Fig Tumour growth after treatment start shown as mean absolute tumour volumes. GOT2-carrying nude mice were treated with (A) 10 MBq ^177^Lu-octreotate (Lu) and/or 125 mg/kg gemcitabine twice weekly (Gem high), and (B) 5 Gy external beam radiotherapy (EBRT) and/or 60 mg/kg gemcitabine twice weekly (Gem low). Error bars show SEM.
(TIF)
