# Supplementary figures and images for: Gemcitabine potentiates the anti-tumour effect of radiation on medullary thyroid cancer
Source: PLoS One. 2019 Nov 14;14(11):e0225260. doi: 10.1371/journal.pone.0225260 (PMC6855663; doi:10.1371/journal.pone.0225260)

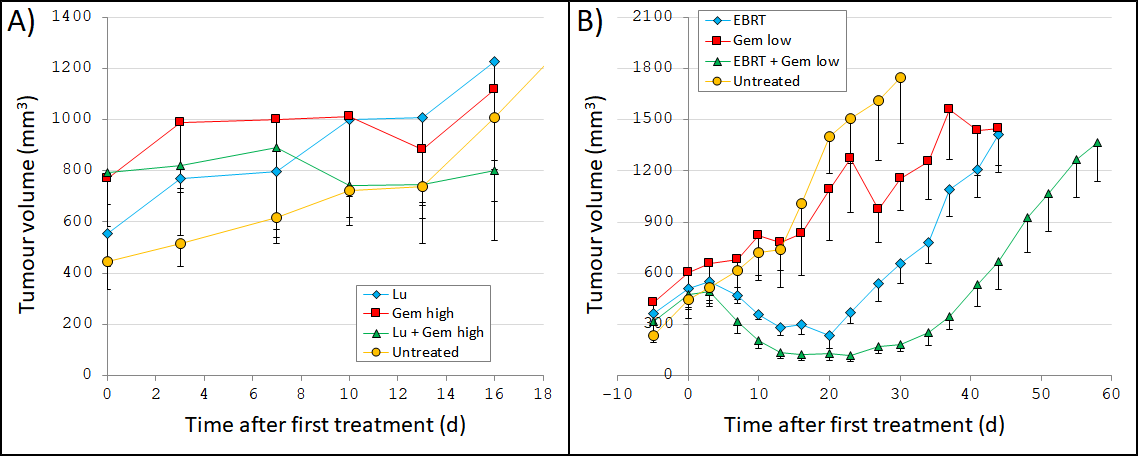

Supplement: S1 Fig — GOT2-carrying nude mice were treated with (A) 10 MBq 177Lu-octreotate (Lu) and/or 125 mg/kg gemcitabine twice weekly (Gem high), and (B) 5 Gy external beam radiotherapy (EBRT) and/or 60 mg/kg gemcitabine twice weekly (Gem low). Error bars show SEM. (TIF) [file pone.0225260.s001.tif]
